# Supplementary material for: Chiroptical activity of hydroxycarboxylic acids with implications for the origin of biological homochirality
Source: Commun Chem. 2021 Jun 10;4:86. doi: 10.1038/s42004-021-00524-z (PMC9814692; doi:10.1038/s42004-021-00524-z)
Supplement: Supplementary file 1 — Supplementary Information [file 42004_2021_524_MOESM1_ESM.pdf]

Supplementary Information

Chiroptical activity of chiral hydroxycarboxylic acids: implications for the origin of biological homochirality

Jana Bocková[1,*], Nykola C. Jones[2], Uwe J. Meierhenrich[1], Søren V. Hoffmann[2] and Cornelia Meinert[1,*]

[1] Institut de Chimie de Nice, University Côte d'Azur, 28 avenue Valrose, 061 08 Nice, France
[2] ISA, Department of Physics and Astronomy, Aarhus University, 8000 Aarhus C, Denmark

*jana.bockova@univ-cotedazur.fr, cornelia.meinert@univ-cotedazur.fr

CONTENT :

Supplementary Figure S1: CD and anisotropy spectra of chiral aliphatic chain hydroxycarboxylic acids in aqueous solution. *page* 2

Supplementary Figure S2: The major photodecomposition mechanisms of aliphatic chain amino acids following $n\pi^*$ excitation of the carboxyl chromophore upon UV irradiation in aqueous solution at a) pH 7 and b) pH 1[1]. *page* 3

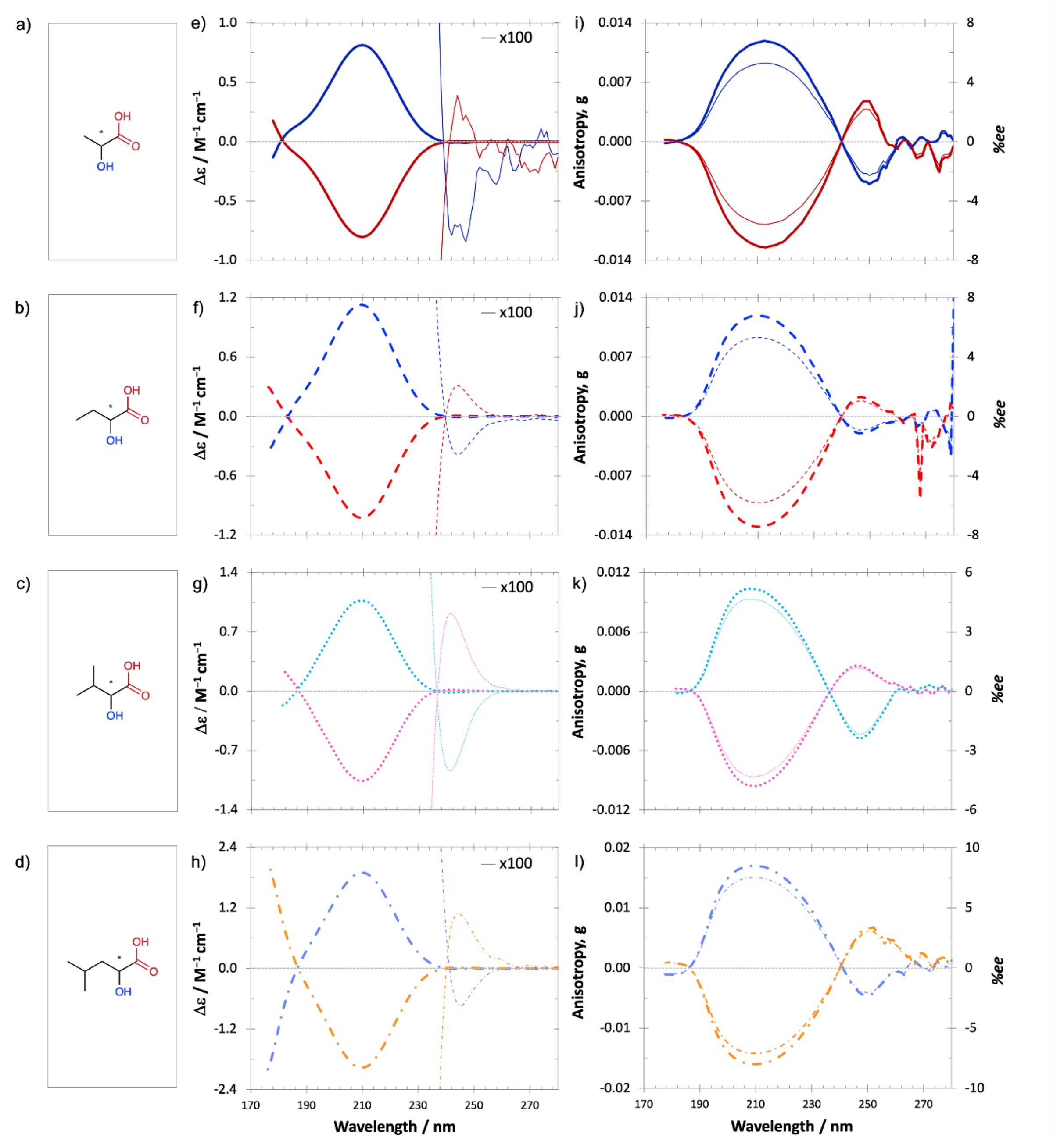

Fig. S1: CD and anisotropy spectra of chiral aliphatic chain hydroxycarboxylic acids in aqueous solution. Schematic structures of a) lactic; b) 2-hydroxybutanoic; c) 2-hydroxy-3-methylbutanoic and d) 2-hydroxy-4-methylpentanoic acid. e) to h) Corresponding electronic circular dichroism spectra of the HCAs in aqueous solution. i) to l) Corresponding anisotropy spectra of the HCAs in aqueous solution (thick) and inducible enantiomeric excess (*%ee)* by either left- or right-circularly polarised light (thin) as a function of wavelength at the extent of reaction ξ = 0.9999 calculated based on relation (1). The D-enantiomers (*R*-) are in shades of red and the L-enantiomers (*S*-) are in shades of blue. Note that due to the uncertainties induced by low absorbance and ECD signals, the values of the anisotropy factor *g* are less reliable above 260 nm for 2-hydroxybutanoic acid, above 270 nm for 2-hydroxy-3-methylbutanoic acid and D-2-hydroxy-4-methylpentanoic acid.

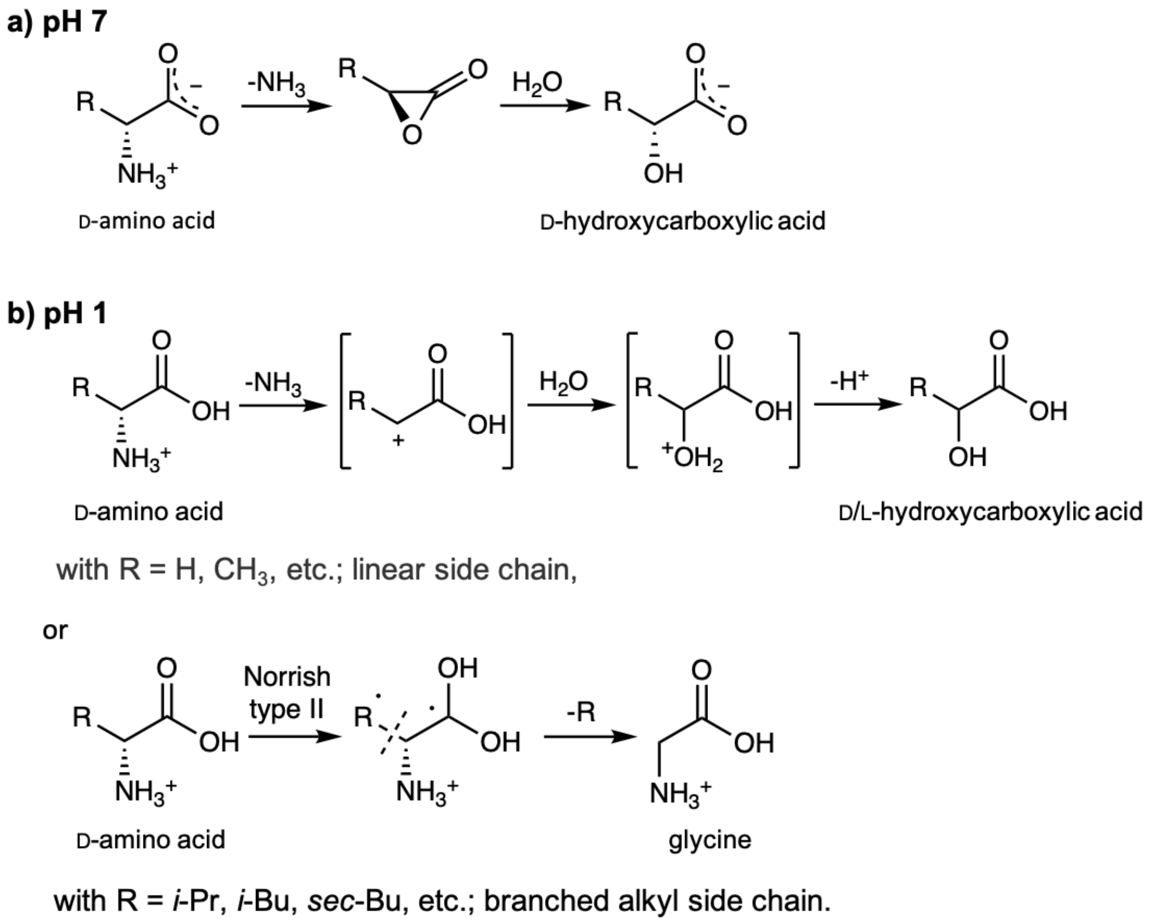

Fig. S2: The major photodecomposition mechanisms of aliphatic chain amino acids following $n\pi^*$ excitation of the carboxyl chromophore upon UV irradiation in aqueous solution at a) pH 7 and b) pH 1[1].

Supplementary References:

1. Nishino, H. *et al.* Absolute asymmetric photoreactions of aliphatic amino acids by circularly polarized synchrotron radiation: Critically pH-dependent photobehavior. *J. Am. Chem. Soc.* **124,** 11618–11627 (2002).
